# Consistent Trait Patterns in a Hyper Diverse Moth Clade Along a Western Himalayan Elevational Gradient

**DOI:** 10.1002/ece3.73083

**Published:** 2026-02-25

**Authors:** Pritha Dey

**Affiliations:** ^1^ Wildlife Institute of India Dehradun India; ^2^ National Centre for Biological Sciences, Tata Institute of Fundamental Research Bengaluru India

## Abstract

Elevation gradients pose significant challenges for flying insects due to temperature and air density shifts, which affect thermoregulation, flight abilities, and distribution patterns. Geometrid moths serve as a useful model for studying these traits. This study investigates how trait patterns in geometrid moth assemblages change across a 1500‐m elevational gradient in the western Himalayas; 697 specimens from 120 geometrid moth species were examined to assess species diversity, turnover, and traits related to body size and flight capabilities. The results show a decline in species diversity with increasing elevation, accompanied by consistent turnover from low to high elevations. However, elevation did not significantly influence morphological traits such as body size (thermal sensitivity) or flight traits like wing loading and maneuverability at the assemblage level. There was high overlap in the trait spaces, thereby showing no significant trait‐space differentiation among individuals across elevations. This possibly suggests that, despite species turnover within assemblages, those with similar ecological roles maintain consistent trait values, contributing to stability in the assemblage‐level trait structure. This study reveals how species traits and assemblage‐level trait distributions vary across an elevational gradient. The overall trait structure remained largely stable across elevations, potentially reflecting abiotic filtering, though direct environmental evidence is lacking. Further, this emphasizes the value of trait‐based approaches to understanding species' responses to environmental changes, especially in paleotropical ecosystems.

## Graphical abstract

This study examines how Geometrid moth assemblages along a 1500‐m elevational gradient in the western Himalayas respond to temperature and air density shifts. While species diversity declines with elevation and turnover occurs from low to high elevations, no significant changes in morphological or flight traits were observed at the assemblage level, suggesting that abiotic factors, rather than competition, drive community traits. The findings highlight the role of trait‐based approaches in understanding species' responses to environmental changes in paleotropical ecosystems.

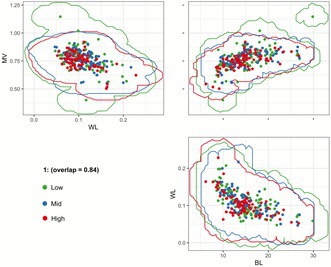

## 1 Introduction

Mountain ecosystems are increasingly threatened by climate change, posing a risk to cold‐adapted and endemic species that depend on these regions (Laiolo et al. 2018; Trew and Maclean 2021). These unique ecosystems show drastic environmental changes over small spatial scales (Rogora et al. 2018), affecting species distributions across gradients. To survive these conditions, species need to adapt physiologically, which is often reflected in their morphological traits (Ryding et al. 2021; Warrington and Waterman 2023).

Morphological traits are critical in defining a species' ecological niche (Violle and Jiang 2009) and by measuring changes in niche space and trait overlap across ecological gradients, we can infer community assembly processes. At lower elevations, warmer temperature and abundant resources reduce constraints, leading to high diversity and niche specialization, with lower trait overlap (Read et al. 2018), as seen in Orthopteran (König et al. 2024) and bee communities (Hoiss et al. 2012). In contrast, higher elevations impose stricter environmental constraints, resulting in lower diversity and higher trait overlap, consistent with the “niche packing” model (Macarthur 1965; Pigot et al. 2016; Pellissier et al. 2018). The degree of trait overlap is shaped by niche differentiation and the displacement of functional trait values over evolutionary time (Macarthur and Levins 1967; Ricklefs and O'Rourke 1975). However, distinguishing traits affected by biotic versus abiotic environmental filters, in trait‐based approaches, can be challenging (Violle and Jiang 2009). Analyzing traits provides a better reflection of adaptation to the environment filters (Garnier et al. 2004; Shipley et al. 2006). Community Assembly Theory (HilleRisLambers et al. 2012; Kraft et al. 2015) predicts that assemblages that are shaped by biotic factors (like interspecific competition) show overdispersed trait spaces, whereas those shaped by abiotic factors (environmental adaptations) are underdispersed, given the relatedness of the species in the assemblage (Weiher and Keddy 1995).

Body size, in particular, is linked to thermoregulation and resource availability, and is a key focus in studies on patterns of traits in various taxa across elevation and latitudes (Chown and Klok 2003; Chown and Gaston 2010). For example, in lizards, higher elevation has higher body size related to higher resource availability, but size decreases with latitude (Lu et al. 2017; Wei et al. 2018; Liang et al. 2023).

Several studies highlight the importance of body size in shaping insect life histories, flight abilities, and ecological interactions (Chakraborty et al. 2021; Outomuro et al. 2021; Darveau 2024). As ectotherms, insects are more susceptible to temperature variations, which means that their body size may be directly influenced by factors like air density, resource abundance, and thermal gradients. For example, bees decrease in size with elevation (Osorio‐Canadas et al. 2022), but increase across latitudes (Gérard et al. 2018). Dragonflies and damselflies are larger in temperate regions than in the tropics (Svensson et al. 2023). Noctuid moths show larger body sizes at higher elevations in temperate regions (Heidrich et al. 2021). However, there is variability within taxa as well. For instance, geometrid moths show diverse patterns: some studies indicate a positive relationship between body size and elevation (Beck et al. 2016, 2017; Brehm et al. 2019), while others reveal no change (Brehm and Fiedler 2004).

Additionally, body size directly impacts flight‐related traits (Grula et al. 2021), with larger insects having higher wing loading and lower maneuverability (Shyy et al. 2016). At higher elevations, lower temperatures favor species with smaller body and reduced wing loading (therefore proportionally long wings) (Dudley 2002), which is advantageous in low air density (Dillon and Dudley 2004; Dillon et al. 2006; Grula et al. 2021).

Morphological traits are, therefore, crucial for our understanding of species assembly (Hoiss et al. 2012; Chichorro et al. 2022). The importance of morphological traits is enhanced while studying assemblages of species‐rich clades such as insects, where species identification is challenging (Yates et al. 2014). Insects adapt to abiotic factors indirectly through interactions with host plants, competitor species, parasitoids, and predators (Hodkinson 2005), or directly through traits like body size, wing size, and colouration (Xing et al. 2018; Bladon et al. 2020; Clusella‐Trullas and Nielsen 2020). Geometridae moths are a good model group to study these traits because this diverse group of moths has been extensively investigated across elevational gradients (Brehm and Fiedler 2003, 2004b; Brehm et al. 2019).

Despite the extensive use of geometrid moths in ecological studies (Brehm and Fiedler 2004; Davis et al. 2013; Beck et al. 2016; Holm, Javoiš, Kaasik, et al. 2019, 2019; Choi et al. 2022; Seifert, Strutzenberger, and Fiedler 2022; Seifert, Strutzenberger, Hausmann, and Fiedler 2022; Araújo Foerster et al. 2024), so far, no study has considered body size and flight‐related traits together to understand the effect of elevation on trait variations in assemblages. By adopting a trait‐based framework, this study aimed to address this gap and contribute to a more detailed understanding of how morphological trait distributions vary along the elevational gradients in geometrid moth assemblages.

In this context, I firstly wanted to understand (1) how do species diversity vary across elevation and how does species turnover within assemblages change along the gradient? (2) do body size and flight‐related traits show a relationship with elevation? and (3) how trait dispersion and the assemblage‐level trait overlap differ along elevations? To address these questions, I studied geometrid moth assemblages across a 1500 m elevation gradient on the fringes of Kedarnath Wildlife Sanctuary. I explored whether diversity declines and compositional turnover increases toward higher elevations, and whether shifts in body size and flight‐related traits correspond to elevation.

Patterns in trait dispersion were also examined to assess whether higher‐elevation assemblages exhibit greater trait overlap, as might be expected under stronger environmental filtering and lower species richness. While these patterns have been observed in bird and bat communities (Schumm et al. 2020; Chakravarty et al. 2023), in the tropical Himalayan mountain system, it remains unexplored if similar patterns apply to insects as well.

## 2 Materials and Methods

### 2.1 Study Area

**MAP 1:**
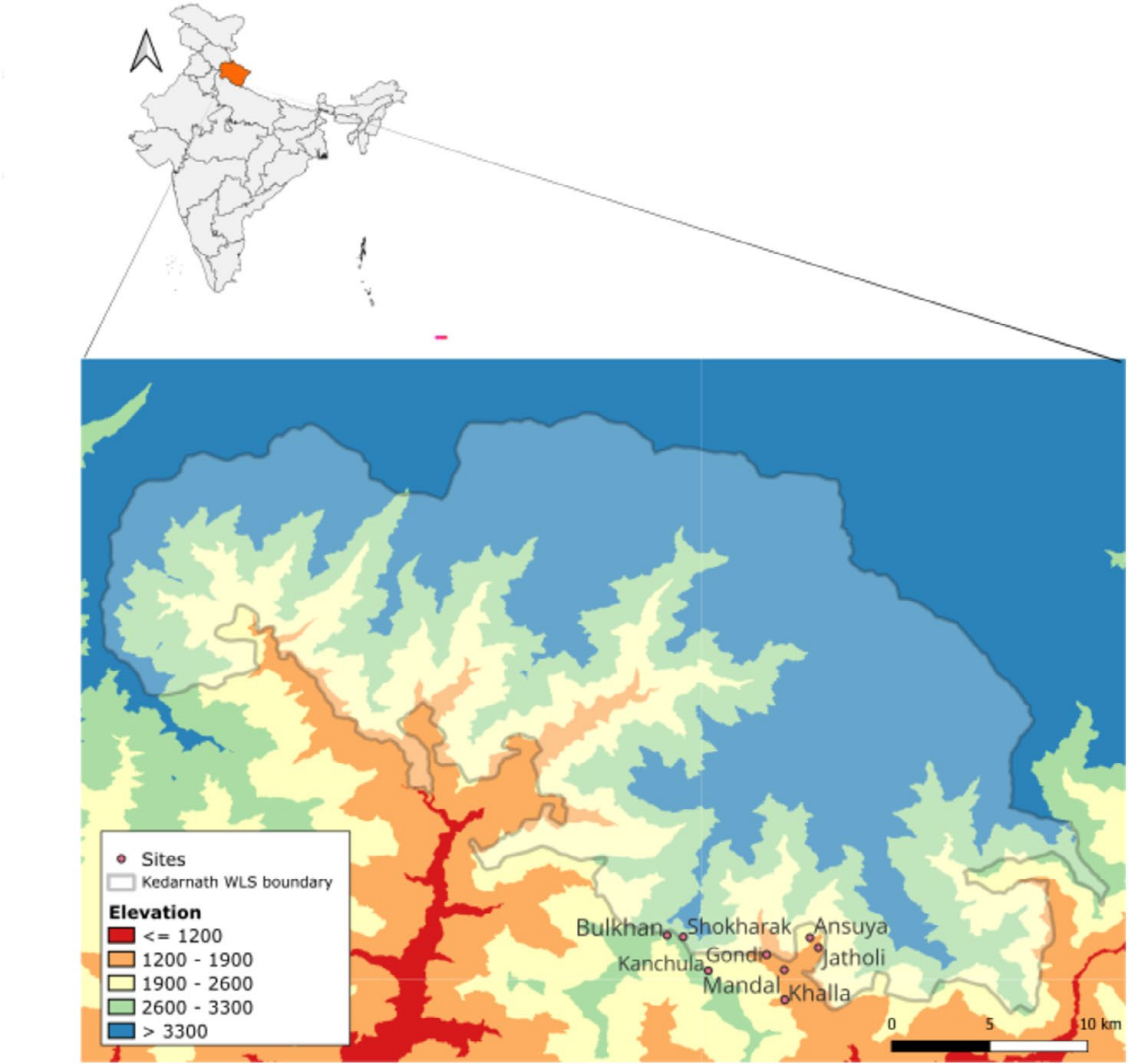
Map showing the sampling locations in and around the buffer zone of Kedarnath Wildlife Sanctuary, which is located in the northern side of the mountainous state of Uttarakhand (highlighted on the map of India on the left side).

The study was done in 2018 around the fringes of the Kedarnath Wildlife Sanctuary (KWLS) (30°25′–30°41′ N, 78° 55′–79°22′ E) located in the Chamoli‐Rudraprayag district in the state of Uttarakhand (Map 1), in Northern India. KWLS covers an area of 975 sq. km and lies in the upper catchment area of the rivers Alakananda and Mandakini, the major tributaries of the river Ganga. The elevation of the protected area ranges from 1160 to 7068 m above sea level, encompassing diverse habitats from moist temperate forests and mixed oak forests to lush alpine meadows. The combination of human pressure, pristine forest areas, and a large altitudinal range makes it an ideal site for biogeographic studies. The forest types are dominated by oak (*Quercus leucotrichophora*, *Quercus floribunda*, *Quercus glauca* , and *Quercus semecarpiflora*). The area receives annual rainfall of about 3000 mm, with maximum contribution during the monsoon (June–September). During the winter months (December–February), heavy snowfall takes place in the alpine and temperate regions (Thakur et al. 2011).

### 2.2 Field Sampling

The study area was stratified based on elevation and vegetation types to sample the moth diversity along the gradients. Sampling was done every 200 m along the elevation from 1500 m up to 3100 m. For analyses, the sampling sites were aggregated into elevational assemblages based on similar elevation and vegetation types (Champion and Seth 1968) as follows:

– Low elevation (< 2000 m): Khalla, Mandal, Gondi, characterized by Himalayan Moist Temperate Forests dominated by *Quercus* spp.
– Mid elevation (2000–2500 m): Jatholi, Ansuya with mixed oak forests;
– High elevation (> 2500 m) Bulkhan and Shokharak, dominated by *Rhododendron* spp.; and Kanchula Kharak, an ecotone site lacking *Rhododendron* but exhibiting vegetation more like higher‐elevation forests than mid‐elevation sites, and thus included in the high‐elevation assemblage.

These elevational assemblages were treated as distinct communities and used as factors in statistical analyses. Replicate sites within each assemblage were selected to represent comparable vegetation structure, which are shown in Map 1.

Moreover, spacing elevation bands out in this manner also reduces the spatial autocorrelation across our sampling sites.

### 2.3 Light‐Trapping Protocol

Vertical light‐sheet traps were used to sample moths. Each light trap consisted of a LepiLED lamp (Brehm 2017) placed in front of a white cloth, which acted as a reflector. This setup is widely used for moth surveys because it maximizes visibility of incoming insects and facilitates standardized sampling. Attracted moths were then hand‐collected directly from the sheet. While hand‐picking does carry some inherent limitations, special care was taken to record all individuals consistently, including geometrids with less conspicuous appearances, thereby minimizing bias.

At each site, 3‐night sampling was done (details provided in Table S1). On each sampling date, two LepiLED light traps were operated for 3–4 h after dusk, depending on the weather conditions (Estimated trap hours‐Trap 1: 1900–2200; Trap 2: 2000–2300 h), which is the peak time of activity of geometrid moths (Brehm and Fiedler 2003; and also from personal observations during reconnaissance survey). Also, logistic constraints do not permit all‐night sampling. Sampling was done for a total ~140 h from March to May (Spring–Summer season) in 2018, during which the average temperatures during sampling were 16.2°C, 11°C, and 7.5°C at the low, mid, and high elevation zones, respectively.

The minimum distance between the two light traps was 50 m, with lamps not in line of sight of neighboring sites, so that cross‐site sampling does not occur. Geometridae moths attracted to traps were counted, and representative samples from each species were collected for morphometric measurements and as vouchers. While many species were represented by only one specimen (singletons), an average of three specimens were collected whenever possible for more reliable morphological analysis. All species were included for morphometric measurements, analyses, and diversity assessment. The specimens were collected only from March to May in 2018, as per the permit from the Forest Department. The specimens were then identified till species, as much as possible. The voucher specimens have been submitted to the Research Collections Facility at the National Centre for Biological Sciences, Bangalore, India.

### 2.4 Morphological Trait Measurement

All voucher specimens were curated and photographed. Morphological traits on the scaled photographs of 697 specimens were measured using the software Image J (Schneider et al. 2012). As different linear measurements widely used proxies for body size in geometrid moths (Brehm et al. 2019; Araújo Foerster et al. 2024), the body length, wingspan (the width between the apices of the forewings, with the trailing wing edge perpendicular to the body), forewing length (measured from the body to the apex of the forewing), and hindwing length were measured. Further, forewing area and hindwing area were used to calculate Wing loading (body length/2*[forewing area *R* + hindwing area *R*]) with body length as a proxy for body mass, and total wing area is twice the area of right forewing and hindwing combined (Shi et al. 2015), and the ratio of hindwing area to forewing area, which is roughly termed maneuverability (Arrizabalaga‐Escudero et al. 2019; Figure S3), which is directly related to the flight capability of moths. These measured traits are thus predicted to govern the occurrence and sustenance of geometrid moths across elevational gradients.

### 2.5 Analyses

#### 2.5.1 Species Diversity

Species identification followed the literature and diagnostic resources cited in (Dey and Hausmann 2021) as being part of the same project. Genitalia dissections or DNA barcoding were not conducted for species delimitation, as the taxonomy of geometrid moths in the study region remains poorly resolved, with limited revisions and reference sequences currently available. Therefore, for the purpose of this study, the specimens were identified to morphospecies based on external morphology, which provided a consistent and reliable framework for examining assemblage‐level patterns of trait variation across elevational gradients.

The elevation bands for the sites selected for sampling in my study area were separated by roughly 200 m each. In order to perform meaningful statistics using elevation as a continuous variable, the bare minimum would have been three sites at every 200 m. This was, unfortunately, not possible given the topography of the landscape. Therefore, the sampling sites were pooled into low, medium, and high, as clarified in the *Field Sampling* section. The low, medium, and high elevations were therefore used as factors. The “iNEXT” R package (Hsieh et al. 2016) was used to calculate rarefied species diversity (Hill number of order *q* = 1, H1 for species diversity) extrapolated to the number of individuals across different elevation categories. This approach allows us to visualize species diversity as a function of sampling effort or the number of individuals sampled. By rarefying the data, the species diversity estimates can be standardized across elevation categories, enabling a comparison of species richness among sites with different sampling intensities. To show sample completeness, the rarefied sample coverage was similarly calculated at each of the elevation categories. A steep slope indicates rapid accumulation of new species with additional sampling effort, suggesting high species richness and/or incomplete sampling. In contrast, a shallow slope indicates that most species have already been sampled, and further sampling is unlikely to yield many new species.

To understand the effect of elevation on the species composition across assemblages, I quantified beta diversity using the incidence‐based Sørensen index, which measures pairwise dissimilarity among sites within the assemblage while accounting for rare species. This approach was chosen to emphasize species turnover rather than abundance differences, as the presence‐absence data provided a more robust comparison across sites with variable sampling effort and low abundances. Beta‐diversity (Sørensen pairwise dissimilarity), and its turnover component (Simpson's pairwise dissimilarity) were calculated as average dissimilarity of the sampling units to their group centroid (Anderson et al. 2006). This analysis was done with the “betapart” and “vegan” packages in R (Oksanen et al. 2009; Baselga and Orme 2012), which decomposes the total beta diversity into two components, (1) Nestedness (βnested)‐ variation in assemblage composition due to species loss or gain across sites that are nested within others, and (2) Turnover (βturnover): variation due to species replacement among sites.

The sampling sites were assigned to three elevational groups—Low, Mid, and High—based on their elevation range and vegetation type, as mentioned earlier in the *Field Sampling* section.

To test whether beta diversity differed significantly among the elevational groups, I used the multivariate dispersion analysis (betadisper function in *vegan* package). This approach measures the average distance between individuals to their group centroid in multivariate space, providing a measure of within‐elevation heterogeneity in species composition.

Differences in beta diversity among the elevation assemblages were assessed using ANOVA on the dispersion values, followed by Tukey's HSD post hoc tests to identify pairwise differences.

#### 2.5.2 Trait Correlations

To examine potential correlations among the morphological traits measured, I first quantified interrelationships among the traits. Pairwise correlations were calculated using Pearson's correlation coefficients. In addition, I conducted a Principal Component Analysis (PCA) on the trait dataset to visualize multivariate trait structure and identify dominant axes of variation.

As many traits are allometrically related to body size, all the linear measurements were size‐corrected by dividing them by body length, as a proxy for body size. The subsequent assemblage‐level analyses were performed on the size‐corrected data set.

#### 2.5.3 Variation in the Body Size and Flight Related Trait Values, Overall Trait Dispersion and Trait Space Overlap

For understanding the effect of elevation on individual traits, two approaches were used. First, community weighted by abundances (standardized effect size; z‐score) mean trait value was calculated for each elevational assemblage (CWM; as used in Lavorel et al. 2008), providing a measure of a mean trait value weighted by the relative abundance of species within that assemblage. Then the raw mean of the individual trait values was compared by pairwise comparisons. It is useful to look at both the approaches as the relationships between community‐weighted mean (CWM) traits and elevation is supposed to reflect selection toward locally optimal traits (Violle and Jiang 2009), but it is limited by the large amount of interspecific trait variation typically found within ecological communities (Muscarella and Uriarte 2016).

To understand the trait dispersion at the subfamily level and at the species assemblage level across the elevational gradient, the homogeneity of dispersion (Anderson et al. 2006) was compared among the individuals in the assemblages in each elevation category. The *betadisper* function of the R package vegan (Oksanen et al. 2009) was used to calculate dispersion, which tests whether the variance or dispersion of two or more groups is significantly different or not based on Euclidean distance. This was followed by a *permutest* for the homogeneity of multivariate dispersion. Additionally, Principal Coordinates Analysis (PCoA) was done to visualize pairwise distances in a two‐dimensional plot followed by PERMANOVA.

To understand the overall trait overlap among individuals in the species assemblages across the elevational gradient, O‐statistic (Read et al. 2018) was used to generate a single community‐level (pooling the assemblages across the elevation) overlap index across multiple traits by fitting nonparametric kernel density functions to the distribution of trait values within an elevation (Mouillot et al. 2005; Geange et al. 2011; Read et al. 2018). For this, body length, wing loading, and maneuverability were chosen as the least correlated independent variable, to avoid multicollinearity. For this, the median pairwise overlap was calculated by first determining the overlap of traits in each species pair, and then taking the median overlap of each species pair in an assemblage. This median overlap value is called the O‐statistic (O for overlap).

The R package *Ostats* (Read et al. 2018) was used to calculate O‐statistic; the multivariate O‐statistic estimation in this package relies on functions imported from the package *hypervolume* (Blonder et al. 2018). To test whether the degree of trait overlap among individuals in each elevational assemblage is greater than or less than expected by chance, the O‐statistic was measured against a null model, where the median of the pairwise hypervolume overlaps is weighted against the abundances of the species pairs in each assemblage or Standardized Effect Size (z‐scores).

## 3 Results

### 3.1 Species Diversity

**FIGURE 1:**
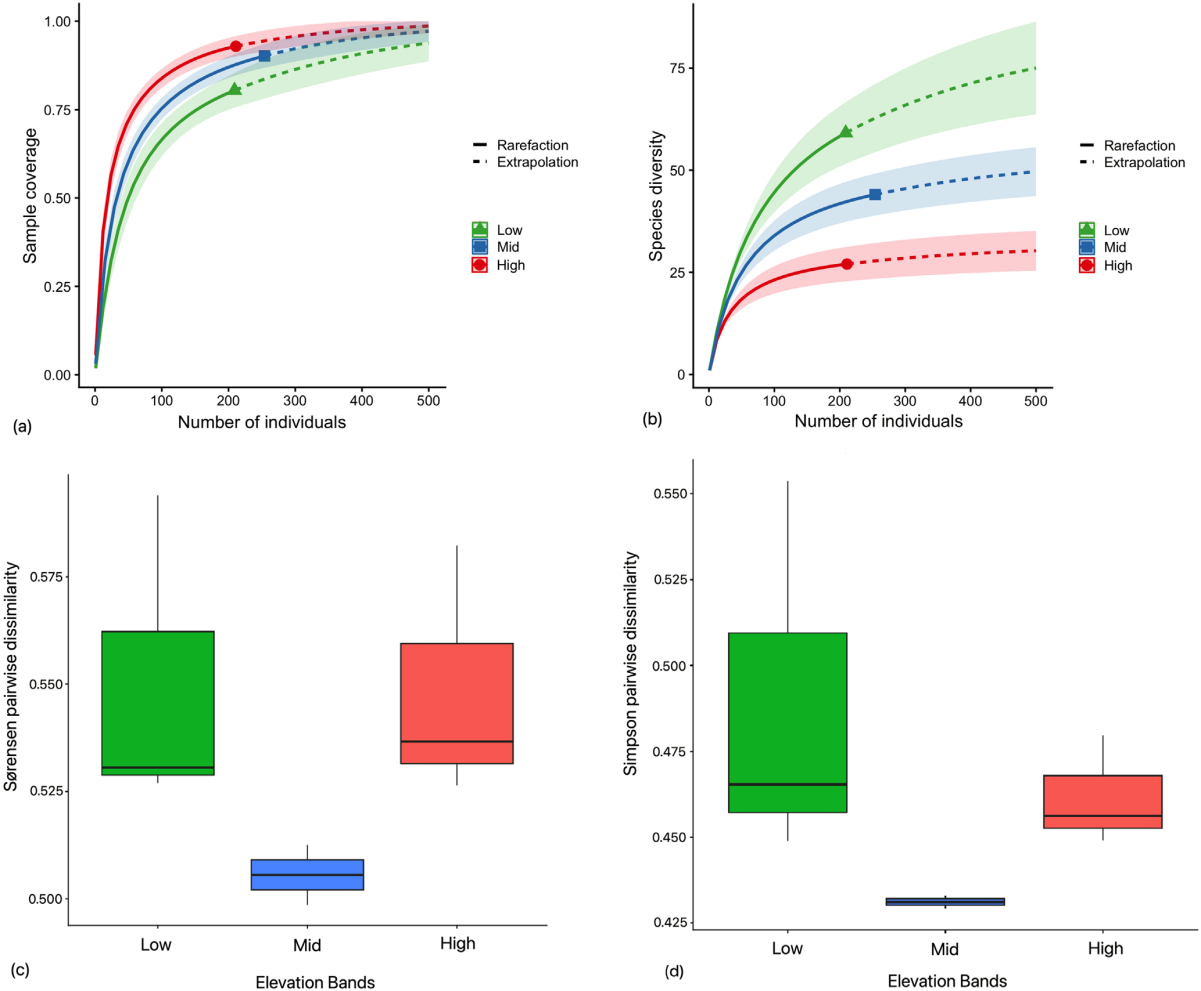
(a) Rarefied curves for sample coverage and (b) species diversity patterns extrapolated across the number of individuals. (c) Total beta diversity among elevational assemblages, calculated as Sørensen pairwise dissimilarity and representing the sum of turnover and nestedness components. (d) Spatial turnover of species composition measured as Simpson pairwise dissimilarity across low‐, mid‐, and high‐elevation assemblages.

Out of 119, there were 56 species‐level identifications, 56 morphospecies identified only till genus, one was identified till subfamily, three morphospecies were identified till tribe and three morphospecies remain unidentified (Table S2). Figure 1b illustrates a decrease in species diversity at higher elevations. Rarefaction curves facilitate the comparison of diversity across various communities or habitats. By scrutinizing the shapes and positions of these curves (Figure 1a,b), the disparities in species diversity, sampling completeness, and overall species diversity along elevation are clear. Figure S1 shows the site‐level comparisons. Venn diagrams were used for visualization of shared and unique species among elevational assemblages (Figure S9) and are not intended as a formal compositional analysis.

Beta diversity of geometrid moth assemblages along the elevational gradient was decomposed into βnested and βturnover values across the different pairs of sites or groups (Figure 1c,d). This visualization helps to understand the relative contributions of nestedness and turnover to the overall beta diversity pattern. The pairwise turnover values ranged roughly from 0.21 to 0.79, indicating moderate to high species turnover within each elevation, whereas the nestedness values were generally much lower (0.008–0.22), suggesting that nestedness contributed relatively little to overall beta diversity within each elevation. Figure 1c shows the total beta diversity among the elevational assemblages, calculated as Sørensen pairwise dissimilarity and representing the sum of turnover and nestedness components; and Figure 1d shows the spatial turnover of species composition measured as Simpson pairwise dissimilarity across assemblages. The mean distances of sites to their group centroids were similar across all three elevation assemblages (Figure S2), with no significant differences (Sørensen pairwise dissimilarity: ANOVA, df = 2, *p* > 0.05; Spatial turnover: ANOVA, df = 2, *p* > 0.05).

### 3.2 Trait Correlations

Several morphological traits were strongly correlated (Figure S7). The correlation matrix revealed high pairwise correlations among linear measurements (e.g., forewing–hindwing length, body length, wingspan, forewing–hindwing area), reflecting allometric scaling. The PCA of the eight traits (Figure S8) indicated that the first principal component (PC1) accounted for 84.8% of the variation and primarily represented flight‐related traits (Maneuverability and Wing loading), while PC2 (8.2%) captured variation in body size‐related traits. These analyses provide essential context for the subsequent assemblage‐level analyses.

### 3.3 Variation in the Body Size‐ and Flight‐Related Trait Values, Overall Trait Dispersion, and Trait Space Overlap

**FIGURE 2:**
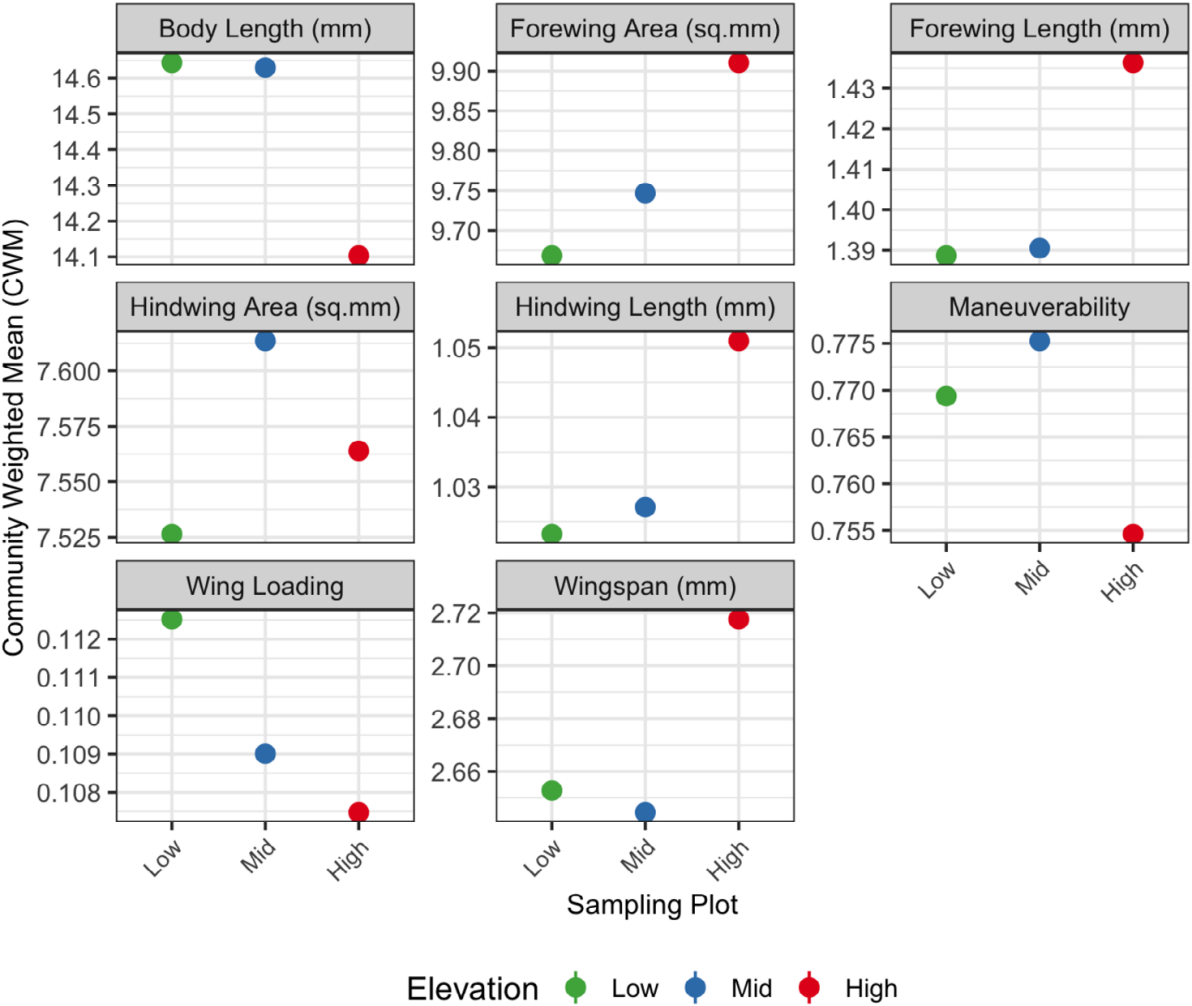
Community‐weighted mean (CWM) values of individual traits across low‐, mid‐, and high‐elevation assemblages. Each point represents the mean trait value for the corresponding elevational assemblage, weighted by species abundance within that community. Since each assemblage represents a single aggregated community, standard errors were not included. These CWMs provide a comparative overview of mean trait values across elevation categories.

The community‐weighted means of the individual trait values show no clear pattern across elevations (Figure 2). Pairwise comparisons of the raw means of the individual traits across the elevation classes also did not show any significant difference (details in Figure S4). However, there is high variability in the CWM at individual sites within each elevation class (Figure S5), indicating that the patterns are sensitive to the abundance of species. Species sharing the same trait values occur across all elevations and vary widely in abundance, which likely obscures elevation‐specific trends. This is further illustrated by the density plots of individual traits, where kernel density estimates show the distribution of each of the traits, indicating the mean values (Figure S6).

**FIGURE 3:**
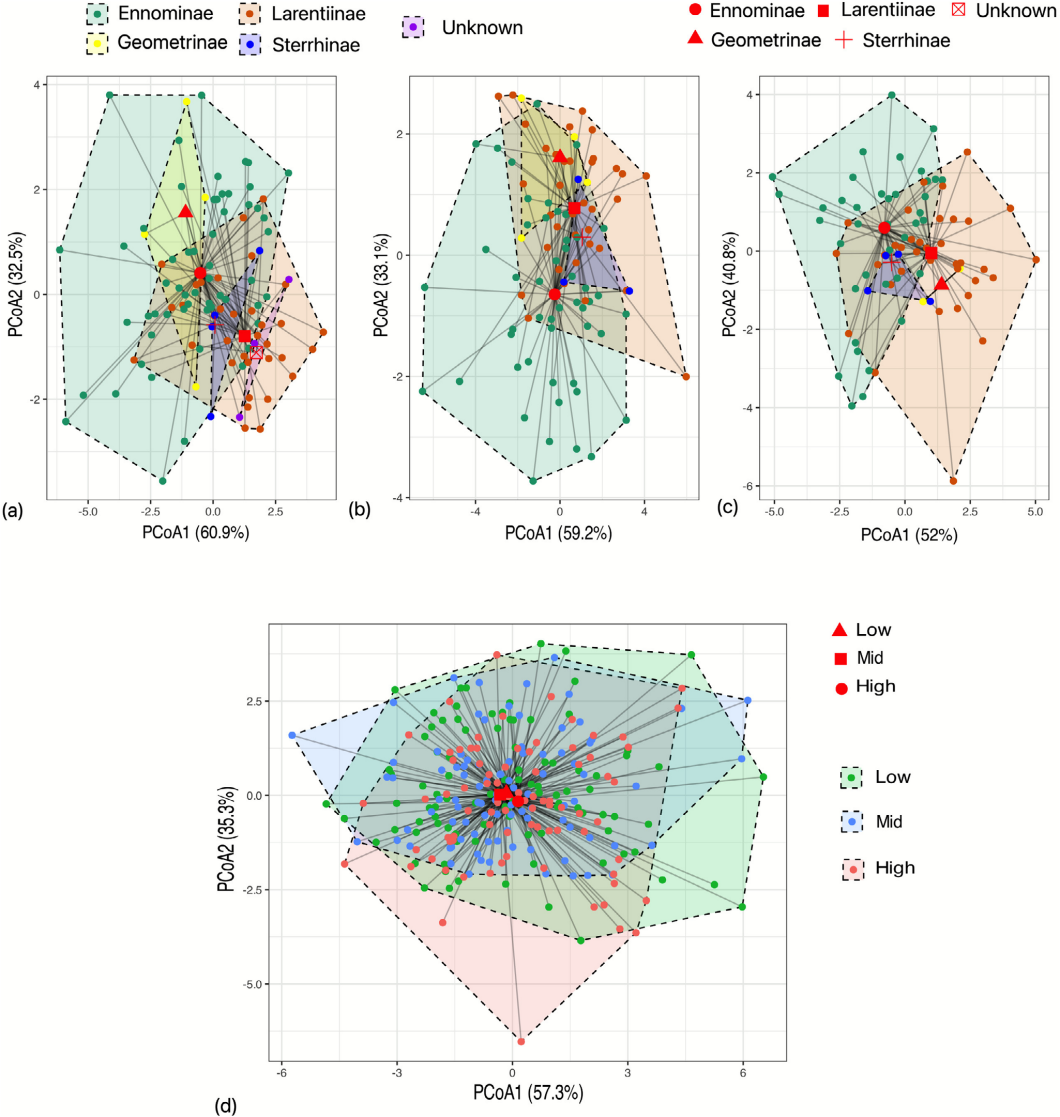
Comparison of subfamily‐level trait dispersion in Geometrid moth assemblages across elevation classes: (a) Low, (b) Mid, and (c) High. Panel (d) shows the overall comparison of trait dispersion at the assemblage level. Each dot represents an individual specimen from the respective assemblage (low, mid, or high elevation). Red–black shapes denote the centroid for each level of comparison. A PERMANOVA was used to test for differences in centroid location among groups, and a test of homogeneity of multivariate dispersion assessed differences in group spread.

Trait dispersion did not differ significantly among the subfamilies in the low (df = 3, *p* = 0.149), mid (df = 3, *p* = 0.252), and high elevation (df = 3, *p* = 0.2). Similarly, no significant differences were found in trait dispersion among assemblages across the elevation (df = 2, *p* = 0.3) (Figure 3).

**FIGURE 4:**
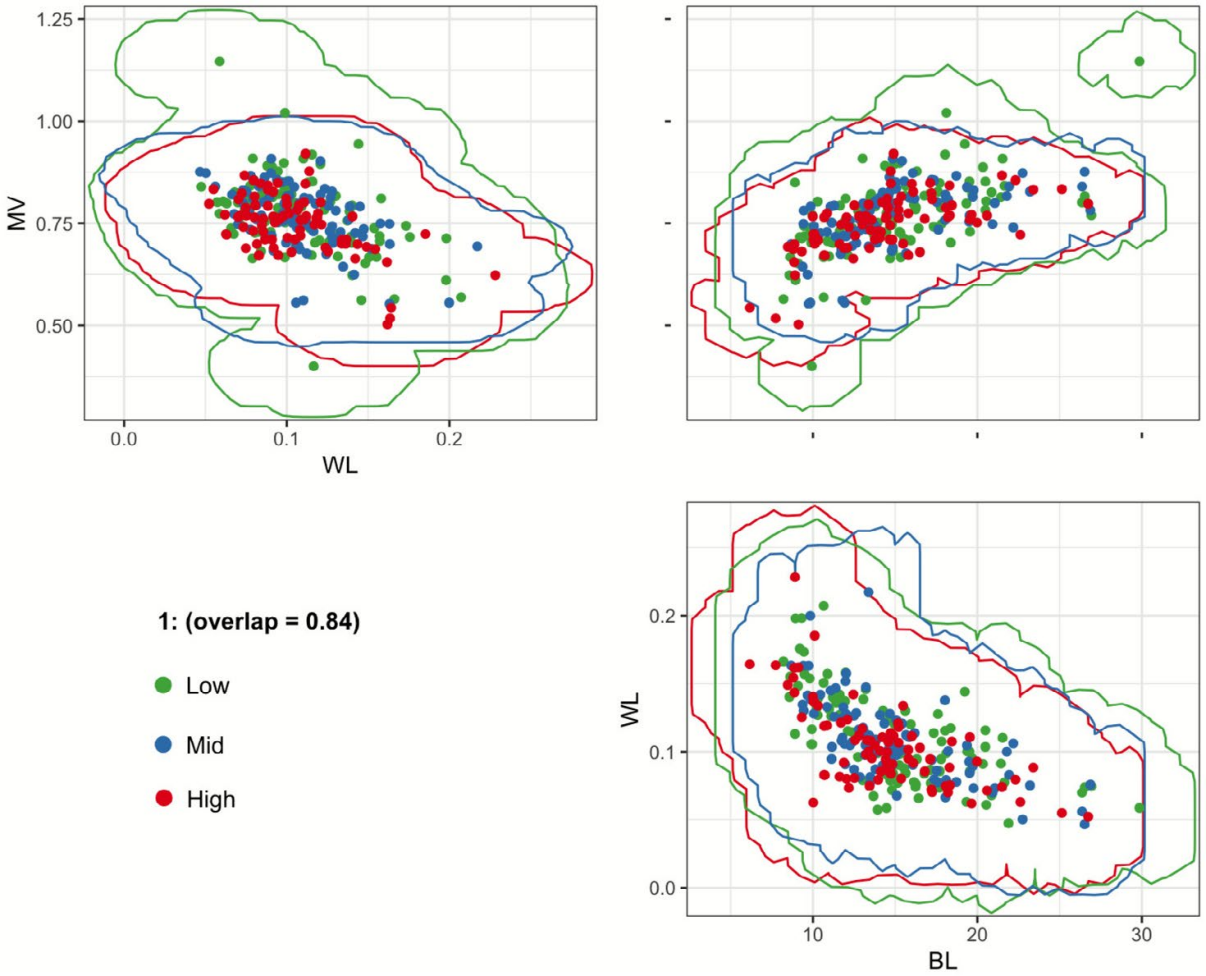
Degree of overlap (O‐statistic) in the multivariate trait space of Geometrid moth assemblages across low, mid, and high elevations. Trait space is based on body length (BL), maneuverability (MV), and wing loading (WL). Each dot represents an individual specimen from the respective assemblage (low, mid, or high elevation).

There is also a high level of trait overlap among elevations for geometrid moth assemblages, as shown by the high (0.84) O‐statistic index of the multivariate trait space (Figure 4).

Together, these results suggest a pattern of species turnover within assemblages along the gradient, while the overall trait structure of assemblages remains largely stable across elevations.

## 4 Discussion

The study describes patterns of trait distribution in geometrid moth assemblages along a 1500‐m gradient in the western Himalaya. The results show a decrease in species diversity with increasing elevation, along with consistent species turnover from the low to the high elevations. However, elevation does not significantly impact morphological traits related to thermal sensitivity (body size) and flight capability (wing loading and maneuverability) at the assemblage level. These findings suggest that geometrid moths maintain trait consistency across different elevations and show no significant trait differentiation among species assemblages across the elevation gradient.

### 4.1 Species Diversity and Turnover Across Elevation

The results indicate a decline in species diversity with elevation, although some species and genera differed in their relative abundance among elevational assemblages. These patterns were not analyzed with trait distributions in this study and therefore only presented qualitatively. For instance, species from subfamily Ennominae, such as from the genera *Alcis* and *Abraxas*, were abundant at low and mid elevations, likely due to their polyphagous diet (Choi et al. 2022), while species like *Dalima truncataria* were less common. In contrast, *Psyra crypta* and several species of the subfamily Larentiinae were more commonly observed at higher‐elevation sites, where Larentiinae species associated with herbaceous vegetation were particularly prevalent.

Within‐assemblage species turnover indicates that sites within the same elevation did not share an identical species set. The abundance of species from the Larentiinae subfamily in the higher elevation sites is similar to trends observed in other mountain gradients (Skou 1986; Brehm and Fiedler 2003; Brehm et al. 2013). Factors such as local host‐plant availability, diet breadth, and breeding strategies also likely affect species diversity (Heidrich et al. 2021; Seifert, Strutzenberger, and Fiedler 2022; Seifert, Strutzenberger, Hausmann, and Fiedler 2022), but these were not analyzed in this study.

The individual light‐trap samples were relatively low; therefore, sites were aggregated into broader elevational assemblages to enable meaningful comparisons. This necessary lumping of sites may have reduced sensitivity to fine‐scale compositional variation among individual sampling locations. I also acknowledge that abundance‐based similarity metrics could provide additional insights into turnover patterns and represent a useful avenue for future work as sample sizes and trait coverage increase.

Despite the observed patterns in species diversity and turnover, assemblage‐level trait distributions related to body size and flight remained broadly consistent across elevations. This suggests that species replacement did not result in detectable shifts in the overall trait‐space along the elevational gradient.

### 4.2 Morphological Trait Variation Across Elevation

Body size significantly affects how insects respond to temperature changes, influencing their distribution and thermal tolerance (Hodkinson et al. 1999; Hodkinson 2005). However, geometrid moths do not show significant body size changes with elevation (Bladon et al. 2020; Heidrich et al. 2021), as we find here as well. This stability in body size may be attributed to their large wings and low wing loading, which enable them to fly across varying temperatures without the need for extensive warm‐up (Casey and Joos 1983; Heinrich 2013). Such adaptations allow geometrid moths to maintain a wide thermal tolerance range, contributing to the consistency of their body size and flight traits across elevations. Similar patterns of adaptation have been observed in other taxa, such as African dung beetles, which also exhibit broad thermal tolerance (Gaston and Chown 1999). This suggests that these moths may prioritize evolutionary trade‐offs that balance thermoregulation and flight capability, rather than finely tuning traits to specific elevational conditions. Furthermore, the high abundance of common species, which are well‐adapted to prevailing environmental conditions, likely reinforces this stability in traits (Cingolani et al. 2007).

The lack of significant morphological trait differentiation may reflect convergence toward similar trait combinations due to the common selective pressures experienced by species across elevations, such as temperature regulation, resource availability, or similar flight demands across forest types. The consistency in traits like body length and wing loading indicates that species occupying different elevations might be functionally equivalent, possibly due to environmental filtering that limits viable morphological strategies under montane conditions. However, the limited number of individuals per sampling site and the aggregation of sites into elevational assemblages may also reduce the ability to detect subtle differences among elevations. Minimal variation in morphological traits along the gradient could reflect the abundance of species with similar traits and evolutionary relatedness (Griffith et al. 2023). Phytophagous insects, including geometrid moths, frequently show conserved traits due to shared host use and evolutionary history (Nylin et al. 2014; Strutzenberger et al. 2017; Slove and Janz 2011; Hardy et al. 2016; Ribeiro and Freitas 2011). This pattern may suggest that geometrid moths in this system are adapted to a range of environmental conditions rather than to specific elevational niches.

Another potential explanation for the stability of traits across the gradient is functional redundancy. Species with similar traits may play comparable ecological roles, which allows for the maintenance of overall trait consistency even as species composition changes (Pillar et al. 2013). While these patterns are consistent across the dataset, sampling constraints warrant caution, as increased sampling effort could reveal additional variation in both species' composition and trait distributions.

### 4.3 Limitations and Future Research

The lack of variation in body size and flight traits in these moths suggests that more definitive conclusions about the mechanisms driving this pattern are constrained by insufficient life‐history, ecological, and abundance data from the Himalayan region. Furthermore, limitations such as restricted sampling to the spring–summer season and the exclusion of lower elevation sites point to the need for further research. Including seasonal variations and additional lower elevation sites could provide a more comprehensive understanding of trait‐environment dynamics. Previous studies from other western Himalayan elevational gradients (Sanyal et al. 2017; Dey 2019) indicate variations in species composition across seasons, highlighting the need for such an investigation. Further, integrating phylogenetic information is essential when examining species traits, their determinants, and their relationships.

Future research could explore the role of life history traits, such as larval host plant use (i.e., diet breadth and host‐plant growth form), seasonal life cycle (i.e., overwintering stage and caterpillar phenology); and predator density in shaping adult body size and flight morphology. Additionally, incorporating genitalia dissections and DNA barcoding would be valuable for improving species delimitation, given the taxonomic uncertainties in the region. Such approaches would strengthen causal understanding of how ecological and evolutionary factors influence trait variation. Long‐term studies tracking potential shifts in trait distributions, under the changing thermal regimes could further reveal how species respond to environmental change and inform conservation efforts to protect the unique biodiversity of the Himalayan ecosystem.

## 5 Conclusion

This study contributes valuable insights into how individual traits, trait variability within assemblages respond to elevational gradients. The lack of clear differentiation in morphological traits overlap across elevations indicates that the trait structure of the assemblages remains relatively stable. While this pattern could be *potentially* consistent with the influence of abiotic filters, we note that no direct environmental variables (e.g., temperature, humidity) were measured in this study. Elevation was used as a broad proxy for environmental conditions, and without explicit trait–environment associations, any inference regarding abiotic filtering remains speculative.

While previous studies from the western Himalayan region, (Sanyal et al. 2017; Dey 2019) have investigated species diversity patterns across elevational gradients, they did not consider trait‐based patterns. Research on moths from this region has generally focused on less diverse groups, such as hawk moths, which showed significant trait variations and internal filters influencing community assembly (Mungee and Athreya 2021). This study, however, focuses on understanding how elevation influences trait‐level patterns among highly diverse geometrid moths, thus contributing new insights to the field.

The absence of significant variation in trait patterns with changing species diversity challenges prevailing notions about the association between elevation and insect morphology. It highlights the possible role of functional redundancy or biotic homogenization (Olden 2006) in maintaining trait distributions across varied elevations. This further underscores the need for a nuanced species‐specific understanding of trait‐environment relationships, particularly as climate change continues to alter the dynamics of this unique ecosystem.

## Author Contributions

**Pritha Dey:** conceptualization (lead), data curation (lead), formal analysis (lead), funding acquisition (lead), investigation (lead), methodology (lead), visualization (equal), writing – original draft (equal).

## Funding

The study was funded by the Rufford Foundation small grants program, UK.

## Conflicts of Interest

The author declares no conflicts of interest.

## Supporting information


**Data S1:** ece373083‐sup‐0001‐TableS1‐S2‐FigureS1‐S9.docx.

## Data Availability

The data is available for reviewers at the following link: https://zenodo.org/uploads/17470539.
