# Supplementary material for: Consistent Trait Patterns in a Hyper Diverse Moth Clade Along a Western Himalayan Elevational Gradient
Source: Ecol Evol. 2026 Feb 25;16(3):e73083. doi: 10.1002/ece3.73083 (PMC12936428; doi:10.1002/ece3.73083)
Supplement: Supplementary file 1 — Data S1: ece373083‐sup‐0001‐TableS1‐S2‐FigureS1‐S9.docx. [file ECE3-16-e73083-s001.docx]

Table S1: Sampling details

| **Elevation class** | **Sites** | **Light Trap plots** | **Elevation (m)** | **#traps (nights)** | **Sampling dates (2018)** | **Season** | **Vegetation Type** | **Total no. of species/site** | **Total no. of individuals collected/site** |
| --- | --- | --- | --- | --- | --- | --- | --- | --- | --- |
| Low | Khalla | P1 | 1633 | 6 traps (3 nights) | 8th April, 8th May, 13th May | Spring-Summer | Himalayan Moist Temperate Forests | 50 | 72 |
|  |  | P2 | 1676 |  | -do- | Spring-Summer | Himalayan Moist Temperate Forests |  |  |
|  | Mandal | P1 | 1617 | 6 traps (3 nights) | 6th April, 12th May, 15th May | Spring-Summer | Himalayan Moist Temperate Forests | 39 | 74 |
|  |  | P2 | 1533 |  | -do- | Spring-Summer | Himalayan Moist Temperate Forests |  |  |
|  | Gondi | P1 | 1638 | 6 traps (3 nights) | 4th April, 9th April | Spring-Summer | Himalayan Moist Temperate Forests | 32 | 77 |
|  |  | P2 | 1620 |  | -do- | Spring-Summer | Himalayan Moist Temperate Forests |  |  |
| Mid | Jatholi | P1 | 1766 | 6 traps (3 nights) | 1st April, 2nd April, 10th May | Spring-Summer | Mixed Oak Forests | 49 | 133 |
|  |  | P2 | 1755 |  |  | Spring-Summer | Mixed Oak Forests |  |  |
|  | Ansuya | P1 | 2035 | 6 traps (3 nights) | 11th April, 12th April,13th April | Spring-Summer | Mixed Oak Forests | 43 | 124 |
|  |  | P2 | 2029 |  | -do- | Spring-Summer | Mixed Oak Forests |  |  |
| High | Kanchula Kharak | P1 | 2646 | 6 traps (3 nights) | 4th May, 6th May, 7th May | Spring-Summer | Sub-alpine forests | 33 | 92 |
|  |  | P2 | 2631 |  | -do- | Spring-Summer | Sub-alpine forests |  |  |
|  | Bulkhan | P1 | 2795 | 6 traps (3 nights) | 23rd April, 24th April, 25th April | Spring-Summer | Sub-alpine forests | 28 | 71 |
|  |  | P2 | 2811 |  | -do- | Spring-Summer | Sub-alpine forests |  |  |
|  | Shokharak | P1 | 3067 | 6 traps (3 nights) | 27th April, 30th April, 1st May | Spring-Summer | Alpine meadows | 20 | 54 |
|  |  | P2 | 3169 |  | -do- | Spring-Summer | Alpine meadows |  |  |


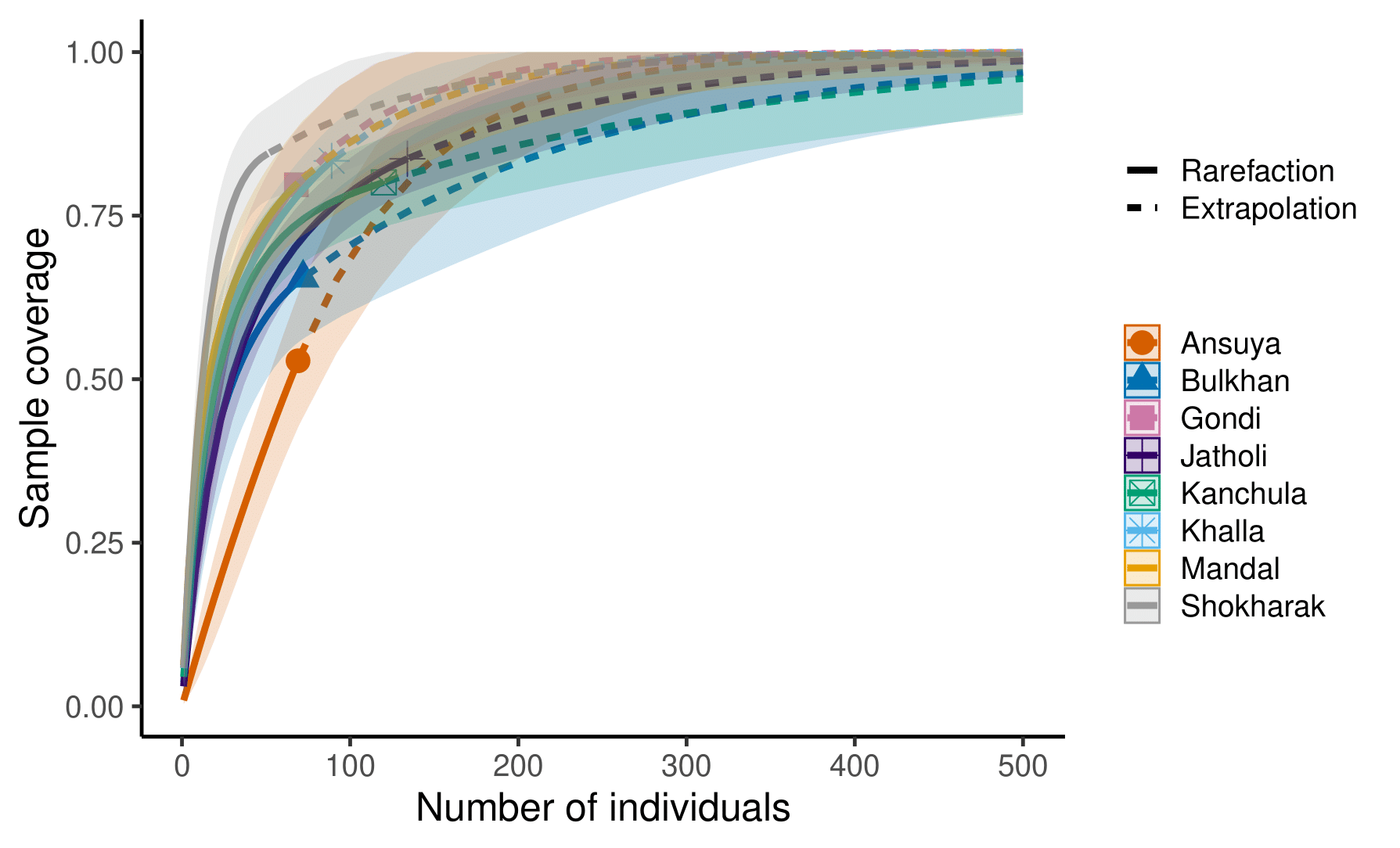

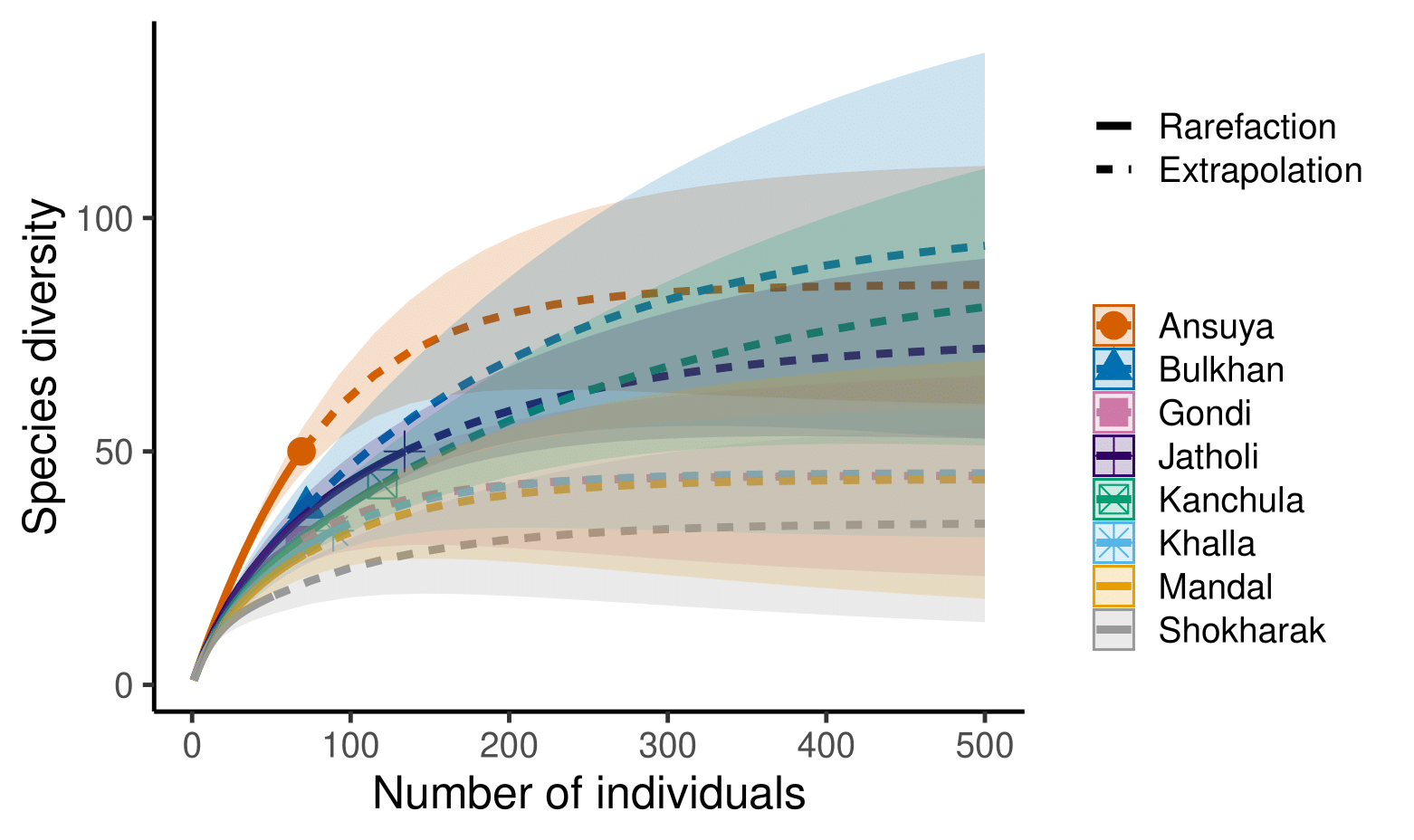


Figure S1: The figure shows the rarefied (left) sample coverage plot and (right) species diversity plots across all the locations, extrapolated to the number of individuals.


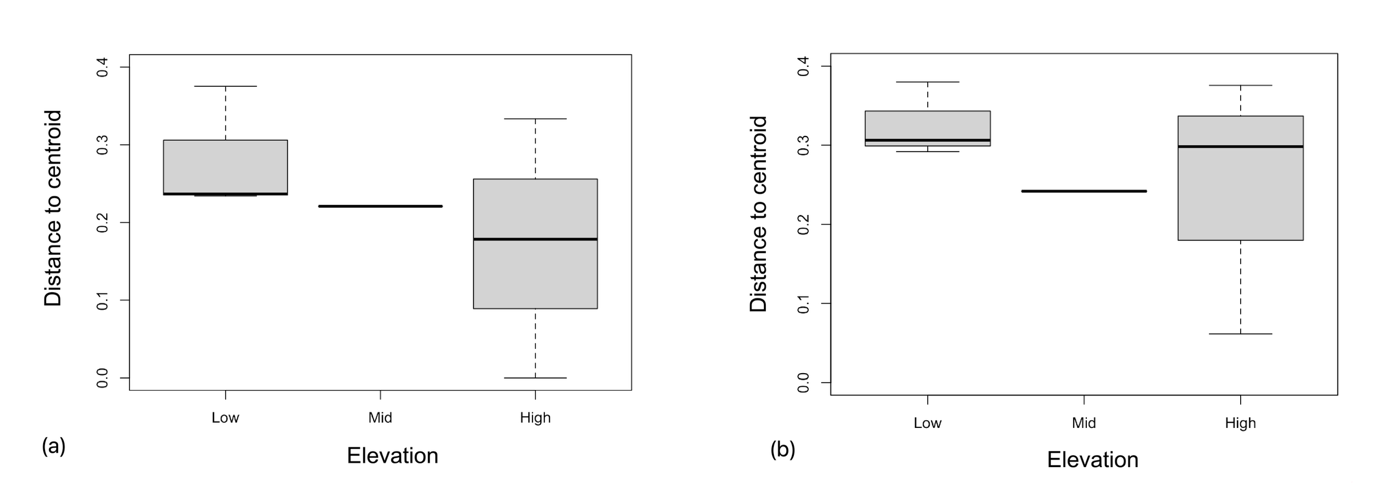


Figure S2: Multivariate dispersion (beta-dispersion) of moth assemblages across elevation bands. Boxplots show the mean distances of sites to their group centroids based on Sørensen pairwise dissimilarity (left) and spatial turnover (right). Distances were comparable among low-, mid-, and high-elevation assemblages, with no significant differences detected (ANOVA, df = 2, p > 0.05 for both metrics), indicating similar within-group compositional heterogeneity across elevations.


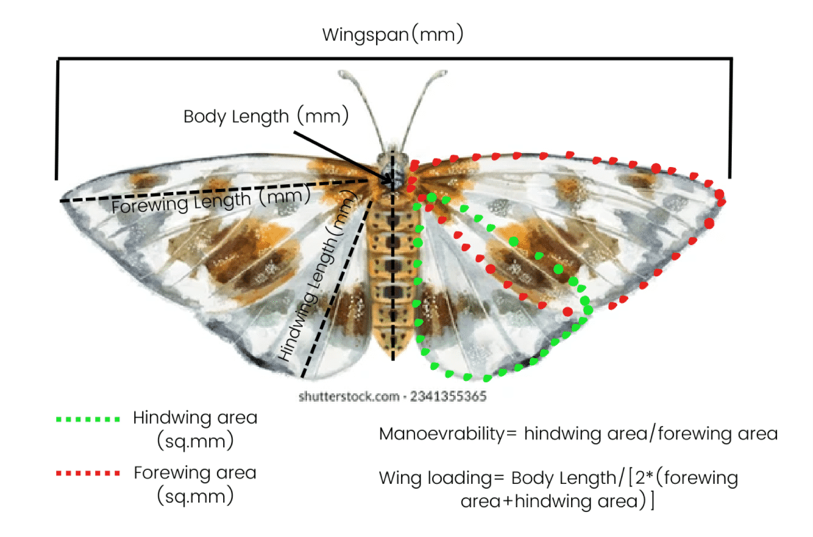


Figure S3: The morphological traits measured from the Geometrid moths in this study


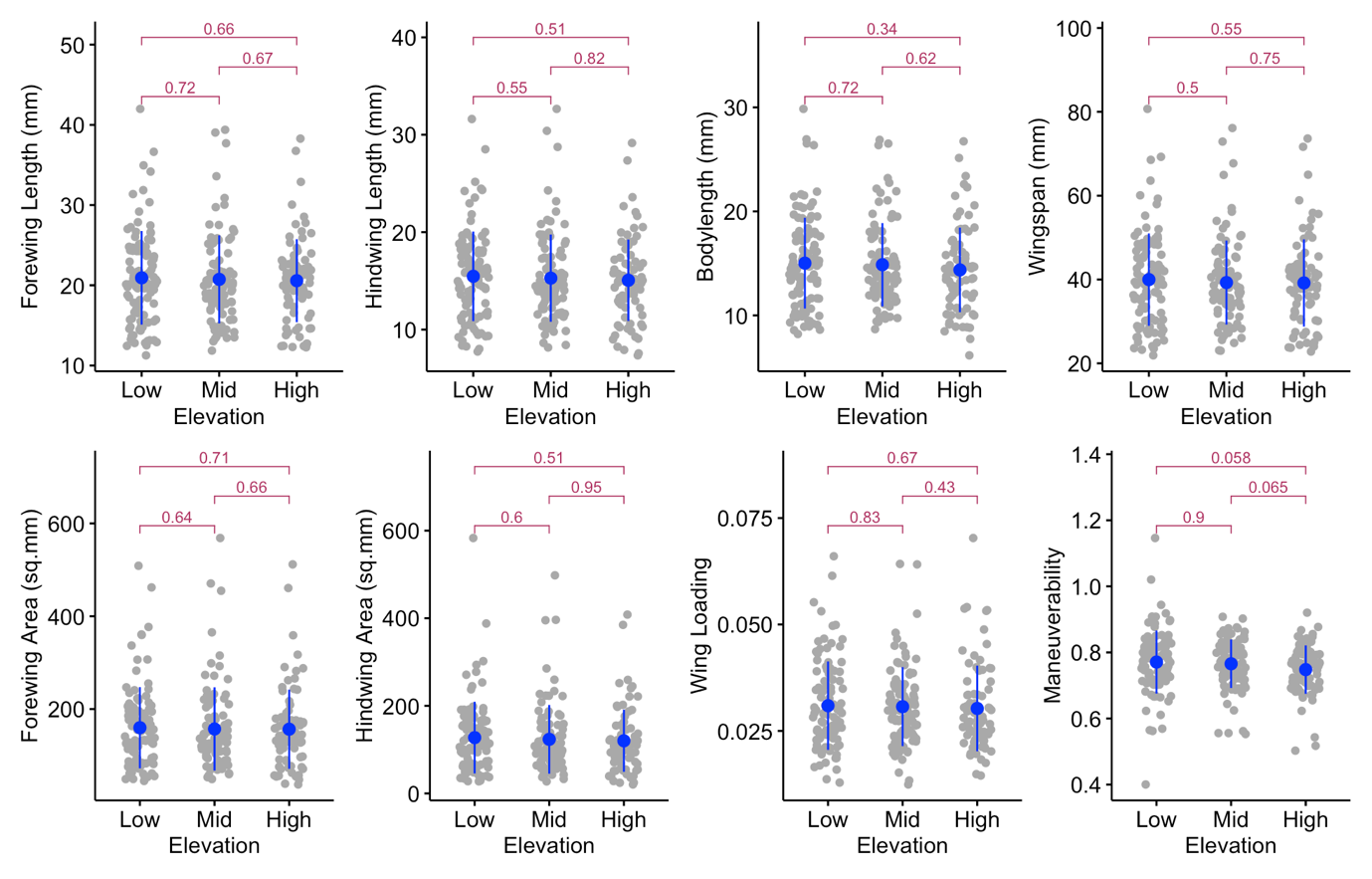


Figure S4: The figure shows (mean ± SE) of each of the traits (in blue) across the low, mid, and high elevation assemblages, with the p-values for pairwise comparisons. The grey dots represent the individuals at each of the elevation assemblages.


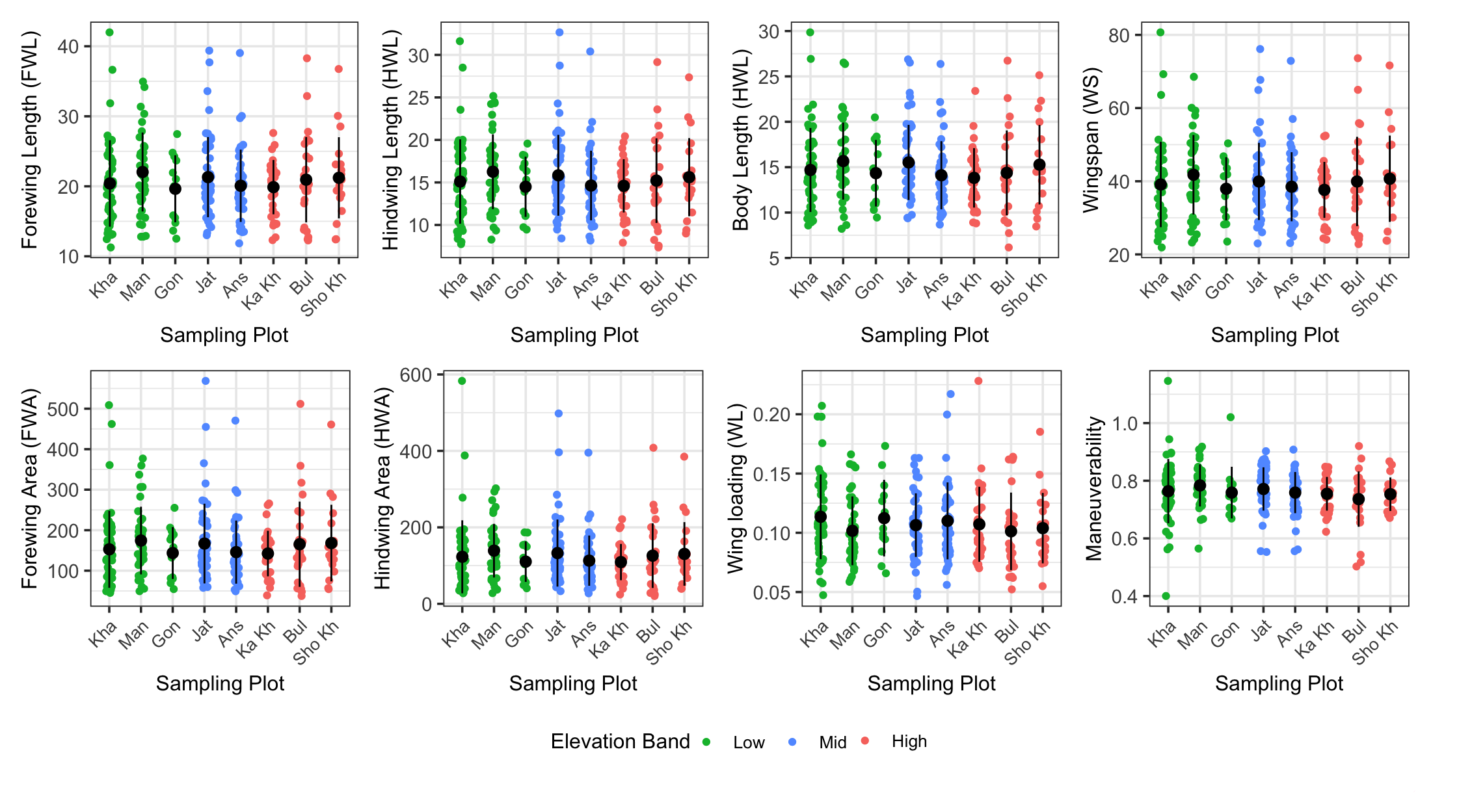


Figure S5The figure shows site-level distribution and (mean ± SE) across elevation. The dots represent individuals at each of the sites. (Site abbreviations: Kha=Khalla; Man=Mandal; Gon=Gondi; Jat=Jatholi; Ans=Ansuya; Ka Kh=Kanchula Kharak; Bul=Bulkhan; Sho Kh=Sho Kharak)


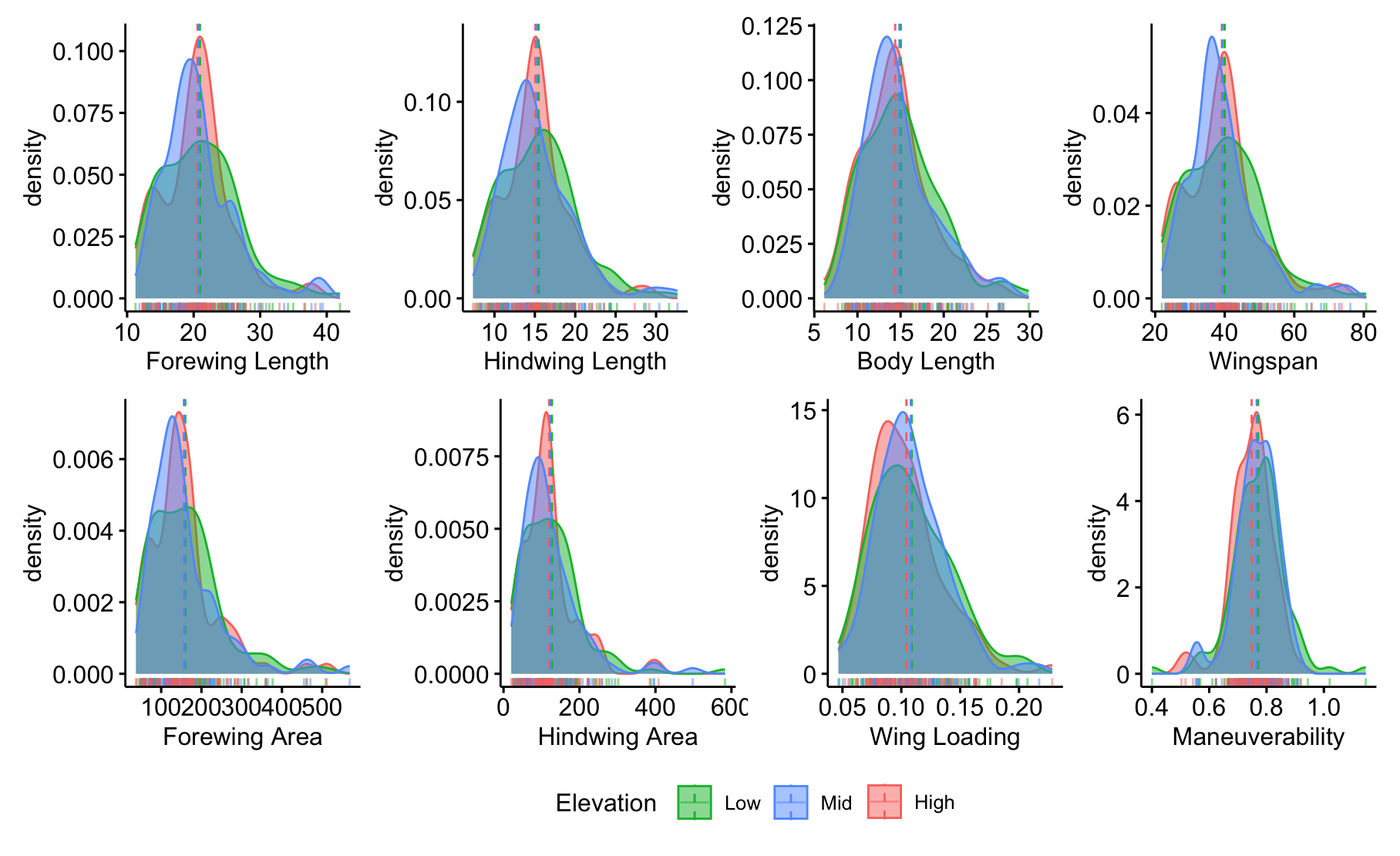


Figure S6: The figure shows the density plots of each of the traits, showing the distribution of the values and the peak shows where the values are concentrated. The dotted line represents the mean of trait distribution across the elevation class


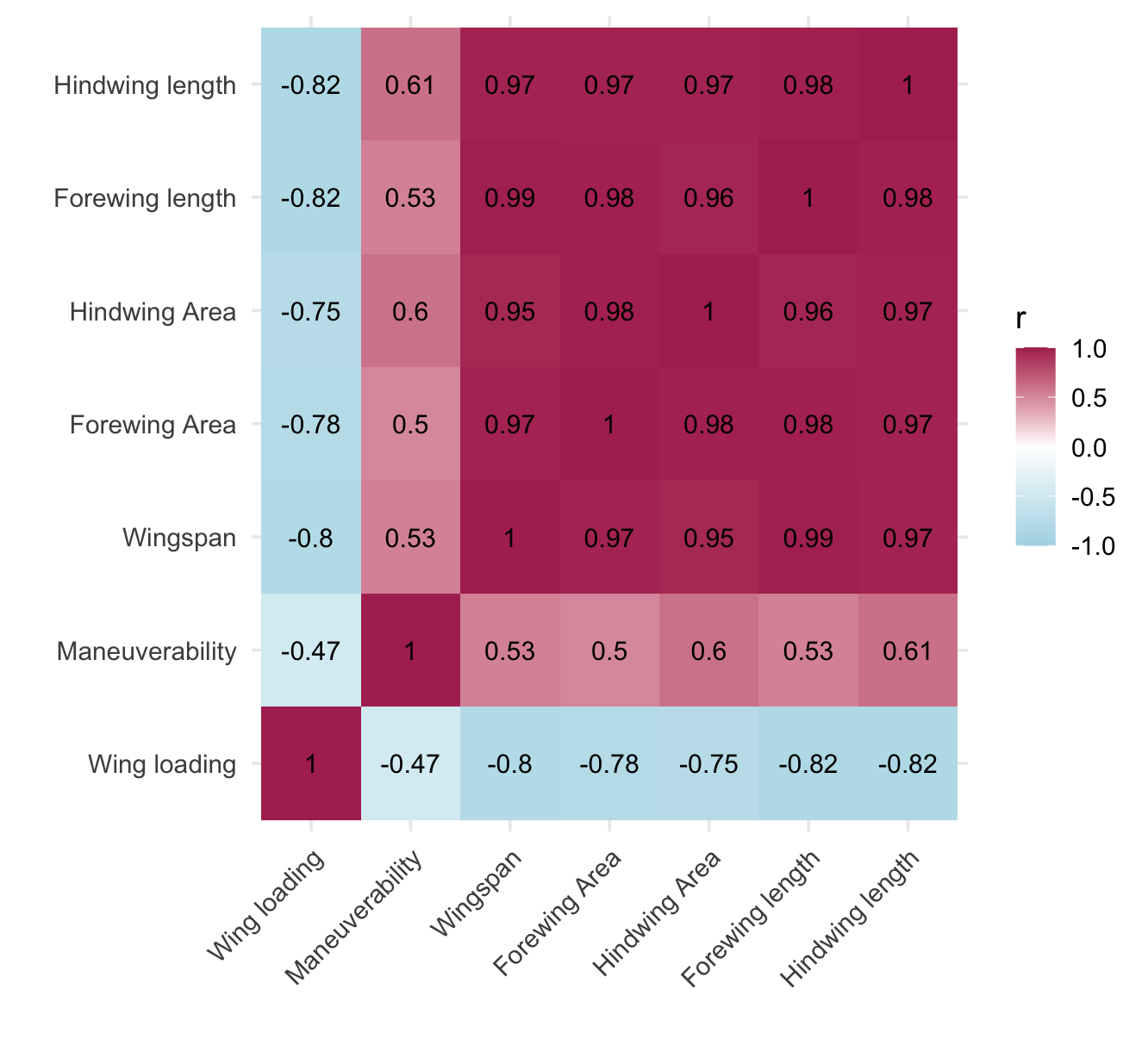


Figure S7: Correlation matrix of the measured traits with the legend showing the r value. This matrix summarizes covariation among traits and highlights sets of traits that tend to co-vary in the assemblages.


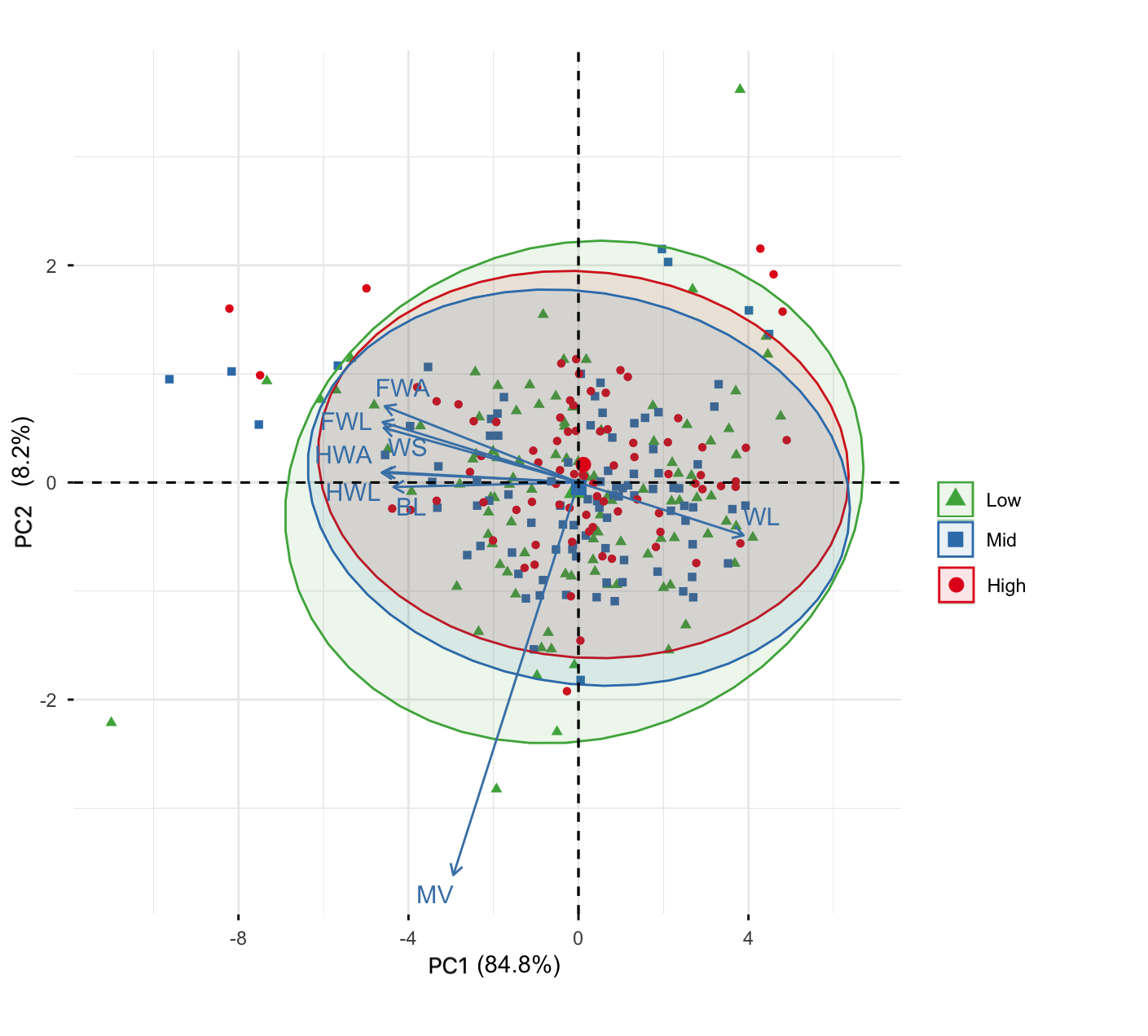


Figure S8: Principal Component Analysis (PCA) of **the measured** trait**s** across elevation groups. Each point represents an individual positioned in multivariate trait space based on the first two principal components. Points are coloured by elevation class and axis labels indicate the proportion of variance explained by PC1 and PC2. This ordination summarizes the major trait gradients underlying assemblage differences across elevations

Table S2: List of species of Geometrid moths in this study with the locations and elevational categories in which they were found

| **Species** | **Sites** | **Elevation** |
| --- | --- | --- |
| Abraxas sp.1 | Khalla | Low |
| Abraxas sp.2 | Ansuya, Kanchula Kharak | Mid, High |
| Abraxas sp.3 | Khalla,Mandal, Jatholi, Ansuya, Kanchula Kharak | Low, Mid, High |
| Achrosis sp. | Khalla | Low |
| Agathia sp. | Khalla,Jatholi | Low,Mid |
| Alcis albifera | Mandal | Low |
| Alcis sp.1 | Khalla,Mandal, Jatholi, Ansuya, Kanchula Kharak,Bulkhan, Shokharak | Low, Mid, High |
| Alcis sp.2 | Khalla, Jatholi, Ansuya, Kanchula Kharak, Bulkhan | Low, Mid, High |
| Alcis sp.3 | Jatholi | Mid |
| Alcis sp.4 | Shokharak | High |
| Alcis sp.5 | Kanchula Kharak | High |
| Alcis-like sp. | Khalla,Mandal, Jatholi, Ansuya | Low,Mid |
| Anonychia grisea | Khalla | Low |
| Arichanna cf. marginata | Khalla, Mandal, Ansuya | Low,Mid |
| Arichanna cf.flavimedia | Jatholi, Ansuya | Mid |
| Arichanna cf.tramesata | Khalla,Mandal, Jatholi, Ansuya, Kanchula Kharak | Low, Mid, High |
| Arichanna sp.1 | Khalla,Mandal, Jatholi, Bulkhan, Shokharak | Low, Mid, High |
| Arichanna sp.2 | Jatholi, Ansuya | Mid |
| Blepharoctenucha virescens | Khalla | Low |
| Chlorodontopera discospilata | Mandal | Low |
| Cidaria sp. | Mandal | Low |
| Cleora sp. | Khalla | Low |
| Comostola cf. hauensteini | Ansuya, Kanchula Kharak | Mid, High |
| Corymica deducta | Khalla | Low |
| Corymica sp. | Jatholi | Mid |
| Craspediopsis bimaculata | Khalla,Mandal,Ansuya,Kanchula Kharak, Bulkhan, Shokharak | Low, Mid, High |
| Ctenognophos cf. eolaria | Khalla,Jatholi | Low,Mid |
| Dalima truncataria | Jatholi, Ansuya | Mid |
| Ecliptopera cf. umbrosaria | Khalla,Mandal, Jatholi, Ansuya, Kanchula Kharak,Bulkhan, Shokharak | Low, Mid, High |
| Ectropis sp. | Mandal | Low |
| Electrophaes cf. fulgidaria | Khalla,Mandal,Ansuya, Bulkhan, Shokharak | Low, Mid, High |
| Electrophaes cf. niveonotata | Jatholi | Mid |
| Electrophaes sp.1 | Khalla, Jatholi, Ansuya, Kanchula Kharak, Bulkhan,Shokharak | Low, Mid, High |
| Electrophaes sp.2 | Jatholi,Bulkhan | Mid, High |
| Electrophaes sp.3 | Khalla,Jatholi, Kanchula Kharak | Low, Mid, High |
| Electrophaes sp.4 | Jatholi | Mid |
| Electrophaes sp.5 | Jatholi, Ansuya, Bulkhan, Shokharak | Mid, High |
| Electrophaes sp.6 | Jatholi | Mid |
| Ennominae 1 | Khalla | Low |
| Eupithecia sp.1 | Khalla, Bulkhan | Low, High |
| Eupithecia sp.2 | Khalla,Jatholi | Low,Mid |
| Eupithecia sp.3 | Mandal,Jatholi, Ansuya, Bulkhan | Low, Mid, High |
| Eupithecia sp.4 | Bulkhan, Kanchula Kharak | High |
| Eustroma melancholica | Ansuya, Shokharak | Mid, High |
| Fascellina plagiata | Jatholi | Mid |
| Fascellina porphyreofusa | Jatholi, Ansuya | Mid |
| Fascellina sp. | Kanchula Kharak, Bulkhan, Shokharak | High |
| Garaeus cf. specularis | Khalla | Low |
| Gasterocome pannosaria | Mid | Low |
| Gnophini 1 | Kanchula Kharak | High |
| Gnophos cf. albidior | Jatholi | Mid |
| Hemithea sp. | Kanchula Kharak | High |
| Herochroma (?) sp. | Mandal | Low |
| Heterolocha sp. | Khalla | Low |
| Heterostegania lunulosa | Bulkhan, Kanchula Kharak | High |
| Heterothera cf. consimilis | Jatholi, Kanchula Kharak | Mid, High |
| Hyalinetta circumflexa | Jatholi, Ansuya | Mid |
| Hypochrosini 1 | Kanchula Kharak | High |
| Hypochrosini 2 | Khalla | Low |
| Hyposidra talaca | Mandal | Low |
| Hysterura cf. protagma | Khalla, Ansuya, Kanchula Kharak, Bulkhan, Shokharak | Low, Mid, High |
| Jodis sp. | Ansuya | Mid |
| Krananda cf. semihyalina | Khalla | Low |
| Lassaba albidaria | Khalla, Jatholi, Ansuya, Bulkhan,Shokharak | Low, Mid, High |
| Leptomiza calcearia | Mandal,Jatholi | Low,Mid |
| Linguisaccus subhyalina | Khalla, Ansuya | Low,Mid |
| Lobogonodes multistriata | Khalla, Gondi, Jatholi | Low,Mid |
| Lomographa sp. | Khalla, Mandal, Gondi, Jatholi | Low,Mid |
| Loxaspilates cf. obliquaria | Kanchula Kharak, Bulkhan, Shokharak | High |
| Luxiaria-like sp. | Mandal, Ansuya | Low,Mid |
| Melanthia catenaria | Mandal | Low |
| Menophra sp. | Mandal | Low |
| Micronidia sp. | Ansuya | Mid |
| Mimomiza cruentaria | Khalla, Mandal, Gondi, Jatholi, Ansuya | Low,Mid |
| Myrioblephara sp. | Mandal, Ansuya, Kanchula Kharak, Bulkhan | High |
| Myrioblephara xanthozonea | Ansuya, Kanchula Kharak, Bulkhan | Mid, High |
| Odontopera bilinearia | Bulkhan | High |
| Odontopera cf.heydena | Kanchula Kharak, Bulkhan | High |
| Odontopera sp.1 | Mandal,Jatholi | Low,Mid |
| Odontopera sp.2 | Shokharak | High |
| Odontopera sp.3 | Jatholi | Mid |
| Odontopera sp.4 | Gondi, Jatholi, Ansuya | Low,Mid |
| Ophthalmitis sp. | Mandal | Low |
| Opisthograptis moelleri | Mandal | Low |
| Orthobrachia flavidior | Ansuya | Mid |
| Orthonama Obstipata | Mandal, Gondi, Jatholi, Bulkhan | Low, Mid, High |
| Parentephria debilis | Bulkhan, Shokharak | High |
| Pasaphila sp. | Khalla | Low |
| Peratophyga hyalinata | Khalla | Low |
| Peratostega deletaria | Mandal | Low |
| Perizoma albofasciata | Shokharak | High |
| Perizoma seriata | Ansuya, Kanchula Kharak, Bulkhan, Shokharak | Mid, High |
| Perizoma sp. | Khalla, Ansuya, Kanchula Kharak, Bulkhan, Shokharak | Low, Mid, High |
| Petelia sp. | Khalla, Mandal, Jatholi | Low,Mid |
| Photoscotosia cf. miniosata | Khalla, Ansuya, Kanchula Kharak, Bulkhan | Low, Mid, High |
| Plagodis inustaria | Jatholi, Ansuya | Mid |
| Plagodis reticulata | Kanchula Kharak | High |
| Plutodes costatus | Khalla | Low |
| Problepsis cf. vulgaris | Mandal | Low |
| Psilalcis sp. | Ansuya | Mid |
| Psyra crypta | Jatholi, Kanchula Kharak, Bulkhan, Shokharak | Mid, High |
| Psyra cuneata | Khalla, Gondi, Kanchula Kharak, Bulkhan,Shokharak | Low, High |
| Rheumaptera sp. | Khalla, Mandal, Jatholi, Kanchula Kharak | Low, Mid, High |
| Rhodostrophia sp. | Gondi, Jatholi, Ansuya, Kanchula Kharak | Low, Mid, High |
| Tanaoctenia cf. haliaria | Khalla, Gondi, Jatholi | Low,Mid |
| Tanaoctenia haliaria | Mandal | Low |
| Tanaorhinus cf. reciprocata | Mandal | Low |
| Trichopterigia sp. | Khalla, Jatholi, Ansuya | Low,Mid |
| Trichopterigia-like sp. | Ansuya | Mid |
| Triphosa sp. | Gondi, Jatholi | Low,Mid |
| Unknown 1 | Mandal | Low |
| Unknown 2 | Khalla | Low |
| Unknown 3 | Khalla | Low |
| Xanthorhoe saturata | Khalla, Mandal, Gondi, Jatholi, Ansuya, Kanchula Kharak | Low, Mid, High |
| Xanthorhoe sp. | Khalla, Mandal, Gondi, Jatholi, Ansuya, Kanchula Kharak | Low, Mid, High |
| Xanthorhoe sp.1 | Khalla, Mandal | Low |
| Xanthorhoe sp.2 | Ansuya | Mid |
| Xanthorhoe-like sp. | Khalla, Gondi | Low |
| Xenoplia maculata | Mandal | Low |
| Xenortholita sp. | Mandal,Gondi, Jatholi,Ansuya | Low,Mid |


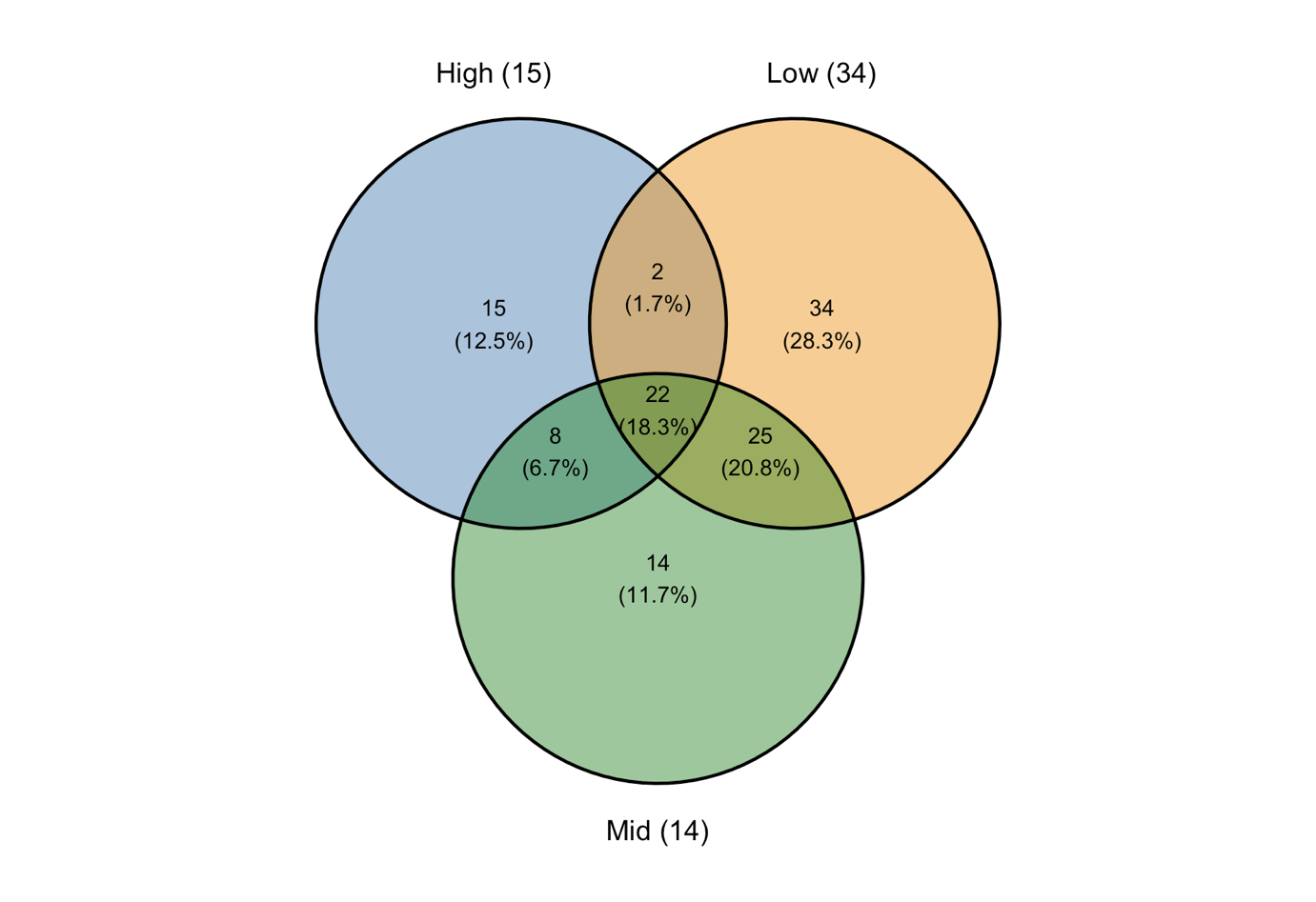


Figure S9: Venn diagram showing species overlap among low-, mid-, and high-elevation geometrid moth assemblages. Numbers in parentheses indicate species recorded exclusively within a given elevational assemblage.
